# Genomic epidemiology of clinical *Campylobacter* spp. at a single health trust site

**DOI:** 10.1099/mgen.0.000227

**Published:** 2018-10-11

**Authors:** Steven J. Dunn, Ben Pascoe, James Turton, Vicki Fleming, Mathew Diggle, Samuel K. Sheppard, Alan McNally, Georgina Manning

**Affiliations:** ^1^​ College of Medical and Dental Sciences, Institute of Microbiology and Infection, University of Birmingham, Birmingham, West Midlands, UK; ^2^​ University of Bath, Bath, UK; ^3^​ University of Nottingham, Nottingham, UK; ^4^​ Nottingham University Hospitals NHS Trust, Nottingham, UK; ^5^​ University of Wolverhampton, Wolverhampton, UK

**Keywords:** *Campylobacter*, epidemiology, clinical, genomics, WGS, United Kingdom

## Abstract

*Campylobacter* is the leading cause of bacterial enteritis in the developed world, and infections with the organism are largely sporadic in nature. Links between sporadic cases have not been established, with the majority of infections thought to be caused by genetically distinct isolates. Using a read-mapping approach, 158 clinical isolates collected during 2014 from the greater Nottinghamshire area were analysed to assess the local population structure and investigate potential case linkages between sporadic cases of campylobacteriosis. Four instances (2.5 %) of case linkage were observed across the dataset. This study demonstrates that case linkage does occur between sporadic *Campylobacter* infections, and provides evidence that a dual multi-locus sequence typing/within-lineage single nucleotide polymorphism typing approach to *Campylobacter* genomic epidemiology provides a benefit to public-health investigations.

## Data Summary

Whole-genome sequencing reads have been deposited in the National Center for Biotechnology Information Sequencing Read Archive under BioProject accession no. PRJNA420922 (url - http://www.ncbi.nlm.nih.gov/bioproject/420922).

**Impact Statement**

*Campylobacter* is the leading cause of bacterial enteritis in the world, and is a significant health and financial burden. Existing research shows that infections with *Campylobacter* spp. are highly genetically diverse, with the majority of studies using core-genome multi-locus sequence typing or whole-genome multi-locus sequence typing approaches. Using a higher-resolution of within-lineage single nucleotide polymorphism based analysis, this study demonstrates linkage between seemingly unrelated clinical cases based on levels of diversity of the causative organism. This study is an important step towards understanding the genetic epidemiology of *Campylobacter*.

## Introduction

*Campylobacter* is the leading cause of bacterial enteritis in the developed world. In the UK, it is responsible for approximately 65 000 illnesses, 22 000 hospitalizations and more than 100 deaths each year – it is also a significant burden to the UK economy, costing tax payers an estimated £900 million per annum [1, 2]. The majority of *Campylobacter* infections are thought to be sporadic in nature and, therefore, do not share a single, commonly identifiable vehicle [3, 4]. The sheer volume of cases that occur make routine genotyping unfeasible for clinical and public-health laboratories and, as a result, infections are reported nationally at the genus level.

The complex epidemiology of *Campylobacter* can be resolved using multi-locus sequence typing (MLST), which assigns house-keeping loci with an arbitrary allele number based on iterative differences in their constituent nucleotide sequences [5, 6]. Query sequences are then compared against a central repository [7] containing previously identified alleles, providing an allelic profile of identified loci, and allowing pairwise comparison of other isolates processed in the same manner. This methodology has been expanded to include additional loci in both core-genome MLST (cgMLST), which assesses a curated set of alleles that are present in >95 % of the species, and whole-genome MLST (wgMLST), which utilises all identified *Campylobacter jejuni* and *Campylobacter coli* loci irrespective of absolute presence [8, 9]. This type of analysis is particularly useful in a clinical environment, as the computational resources required are significantly lower than alternative approaches (e.g. read mapping), and the results are comparable and reproducible across laboratories [8–10].

Whilst useful in the clinical environment, MLST methods rely on *de novo* assemblies, which excludes relevant genomic data present in whole-genome sequencing reads. Assemblies are consensus sequences derived from the statistically informed merging of raw sequence reads [11, 12]. Reads may cover any given position within a genome multiple times; however, that coverage is collapsed to 1× during assembly due to the generation of contigs. Read mapping confers a significant increase in the number of queried alleles at a genomic position, increasing the accuracy and quality of identified variants, and as such confers a finer scale of resolution to epidemiological investigation [13, 14]. Additionally, a major difference in resolution between read mapping and cgMLST lies in the choice of reference – using a highly similar reference to map reads against (e.g. from the same sequence type) permits a greater range of comparison compared to the cgMLST scheme, which is based on the core of the genus and as such only identifies variants at a limited number of loci. Read-mapping approaches have been used to resolve a number of high-profile epidemiological cases [15–17], and can be used in combination with a first-pass analysis using typing schemes such as wgMLST or cgMLST to provide additional scrutiny.

This study aimed to investigate case linkage between clinical *Campylobacter* isolates across the greater Nottinghamshire area. By utilising alternative whole-genome methodologies (i.e. read mapping) in combination with existing comparative tools, additional fine-scale associations might be apparent that would be undetectable when relying solely on other methods.

## Methods

### Isolates and culture

Isolates were collected from Nottingham University Hospitals NHS trust, Queen's Medical Centre (QMC) hospital over a defined 1 year sampling period from January 1st to December 31st 2014. An approximate, raw number of isolates to be randomly sampled each month was determined using local historic incidence data supplied by QMC, with an aim of sampling a representative cohort of approximately 25 % of the total number of *Campylobacter* spp. isolated by the hospital each year.

All isolates were collected from QMC and transported on ice, and stored at −80 °C using Microbank Preservation Beads (Prolab Diagnostics). Isolates were cultured directly onto Campylobacter blood-free selective agar base (Oxoid), supplemented with 16 mg cefoperazone per 500 ml and 5 mg amphotericin B per 500 ml (Oxoid). Culture plates were incubated for 48 h at 37 °C in an anaerobic workstation (Don Whitley Scientific), configured with a microaerobic gas canister (5 % O_2_, 10 % CO_2_ and 85 % N_2_; BOC). Samples that failed to produce sufficient growth were re-cultured in Mueller–Hinton broth (Oxoid) also supplemented with selective formula (Oxoid).

### DNA extraction and whole-genome sequencing

DNA was prepared for whole-genome sequencing using a QIAmp DNA mini kit (Qiagen). DNA was quantified using the Qubit fluorometric platform (ThermoFisher Scientific) and quality assessed using the Nanodrop 2000 (ThermoFisher Scientific). Samples that did not have absorbance ratios between *A*_260_ : *A*_280_ 1.80–2.00 and *A*_200_ : *A*_220_ 2.00–2.20 were discarded. Genomic libraries were prepared using Illumina’s Nextera XT kit, as per the manufacturer's protocol (Illumina), and sequenced using the Illumina MiSeq platform and V3-600 reagent cartridge to generate 2×250 bp paired-end reads.

### Genomic analysis

Reads were assembled using SPAdes 3.5.0 [11]. MLST profiles providing data on clonal complex (CC) and constituent sequence types were obtained using the PubMLST *C. jejuni/C. coli* allele database hosted on the BigsDB platform [7]. Isolates that identified multiple alleles of housekeeping genes were excluded from analysis as they may potentially represent a mixed sample. Whole-genome assemblies were analysed using Quast [18]. The final genomic dataset (*n*=165) had a mean N50 (i.e. 50% of the genome is in contigs of this length or greater) of 120 954 bp [95 % confidence interval (CI): 96 616 to 145 291 bp), a mean total length of 168 001 bp (95 % CI: 1 666 466 to 1 693 696 bp) and a mean number of contigs of 169 (95 % CI: 129.4 to 208.6). Assemblies were annotated using Prokka [19] in conjunction with a custom database constructed from the Gundogdu reannotation of NCTC 11168 [20].

The core-genome phylogeny of assembled genomes was reconstructed using the Parsnp tool from the Harvest software suite [21]. *C. jejuni* and *C. coli* isolates were processed separately, with the datasets comprising 153 and 12 isolates, respectively. The resulting newick formatted trees were visualized using the Interactive Tree of Life (iTOL) [22] and used to infer genotypic similarity. Isolates that shared the same clade with zero branch length (i.e. no variation at the core-genome level) were treated as potentially linked, with additional information such as matching CC and sequence type used to qualify further investigation. Due to the overall phylogenetic distance within the *C. jejuni* dataset, additional phylogenies were reconstructed for individual CCs displaying potential case linkages – increasing the region assigned as ‘core’ by Parsnp and further increasing the comparative resolution.

Isolates that were homologous at the core-genome level were compared using Snippy 4.0-dev2 (based on Freebayes), with the *de novo* assembly containing the greatest number of loci (as defined using BigsDB) used as a reference and the reads from the isolate identified as homologous used to query [23]. The resulting polymorphisms were filtered to only include sites with a base and mapping quality of ≥Q30, a minimum read depth of 8 and a minimum fraction (i.e. presence amongst total reads) of 90 %. Variants that showed signatures of recombination based on spatial proximity and density in the mapping analysis were also removed. Clonal-complex specific phylogenies were compared using the BigsDB platform’s Genome Comparator module to obtain whole-genome MLST profiles [7]. A cut off value of ≤20 variable loci in a pairwise comparison was used to indicate linkage [9]. Truncated loci (e.g. at the ends of contigs) or those that were unique to an isolate’s assembly were discounted from the comparison.

## Results and Discussion

**Fig. 1.**
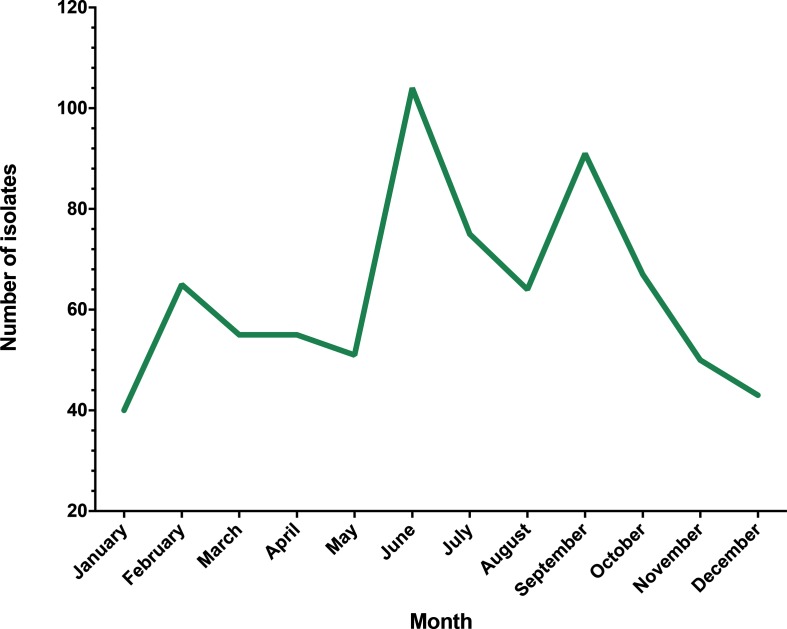
Gross monthly incidence of clinical *Campylobacter* spp. infections collected in Nottingham during 2014. Isolates were collected at the QMC, a large hospital servicing the greater Nottinghamshire area, with an effective collection date ranging from January 1st to December 31st 2014.

During 2014, 760 eligible clinical *Campylobacter* spp. isolates were collected at the QMC (Fig. 1), with the greatest incidence occurring in June (*n*=104). From this, 185 isolates (24 %) were selected for whole-genome sequencing. Sequences (*n*=17) were discounted from analysis due to contaminant reads, being mixed or sequencing errors that resulted in inadequate assemblies or genome coverage (~20×).

### Population structure of Nottinghamshire clinical isolates mirrors that of the UK

**Fig. 2.**
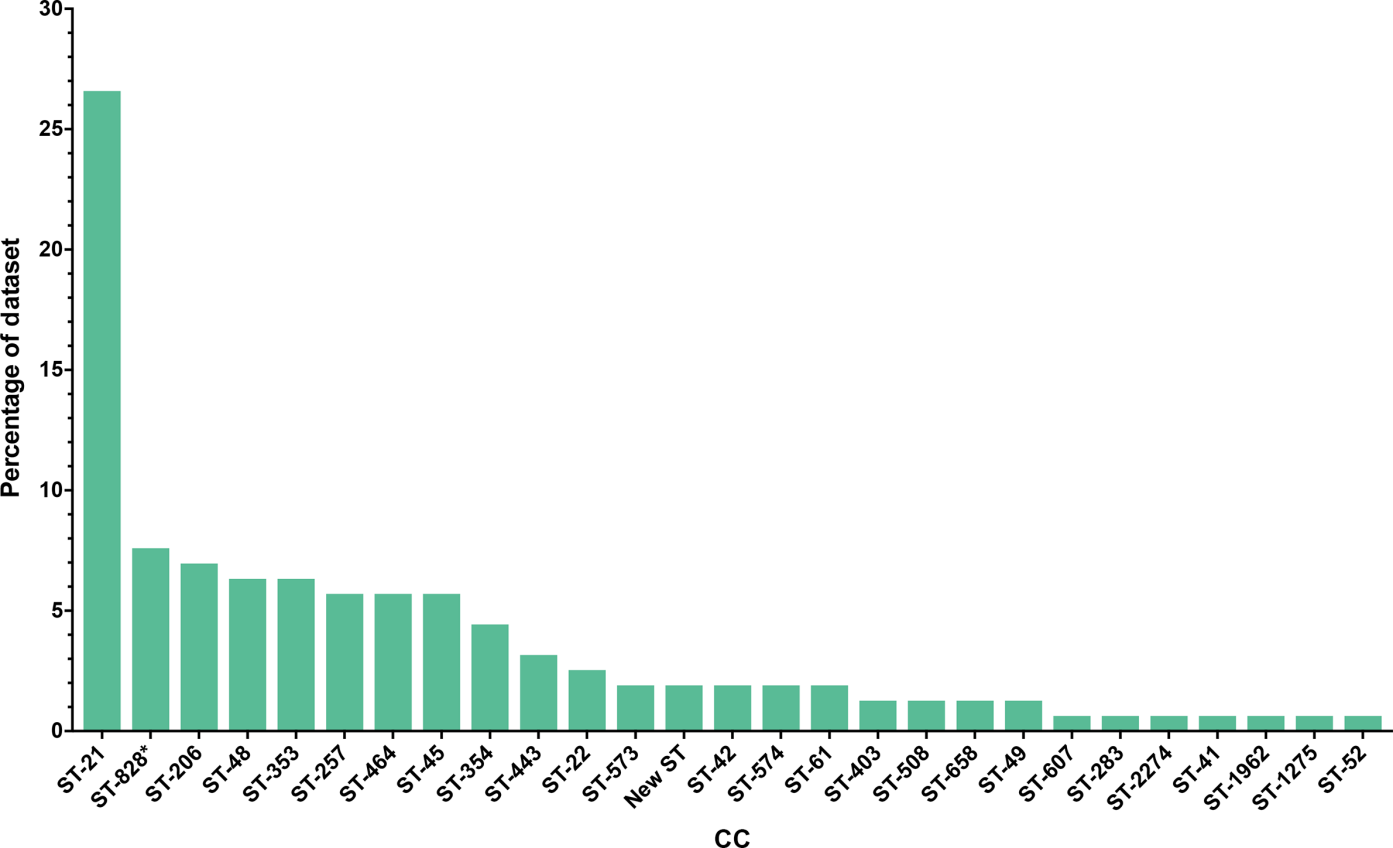
Distribution of CCs amongst 158 clinical *Campylobacter* spp. isolates obtained during 2014 from QMC, Nottinghamshire, UK. These isolates represent 20.8 % of all *Campylobacter* spp. isolates collected at the QMC during 2014 (*n*=760). *, CC ST-828 represents isolates belonging to *C. coli*.

A total of 168 draft genomes were produced with sufficient quality and coverage depth for use in further analysis, revealing isolates belonging to a total of 29 unique CCs (Fig. 2). Of these 168 isolates, 91 % (*n*=153) belonged to *C. jejuni* subsp. *jejuni* and a further 7.1 % (*n*=12) were identified as belonging to *C. coli* CC ST-828. The remaining 1.9 % (*n*=3) isolates were not assigned to an existing CC.

The distribution of CCs amongst the sequenced samples was similar to other parts of the UK [8], with ST-21 the major prevalent CC in the dataset, accounting for 26.5 % (*n*=42) of all isolates. A further seven CCs contained more than 5 % of the total dataset (ST-828, ST-206, ST-48, ST-353, ST-257, ST-464 and ST-45). The distribution of these major CCs was identical to that of a larger published dataset encompassing isolates from Oxfordshire, Scotland and North-West England [8], indicating that the *Campylobacter* population in Nottingham is comparable to the UK as a whole.

**Fig. 3.**
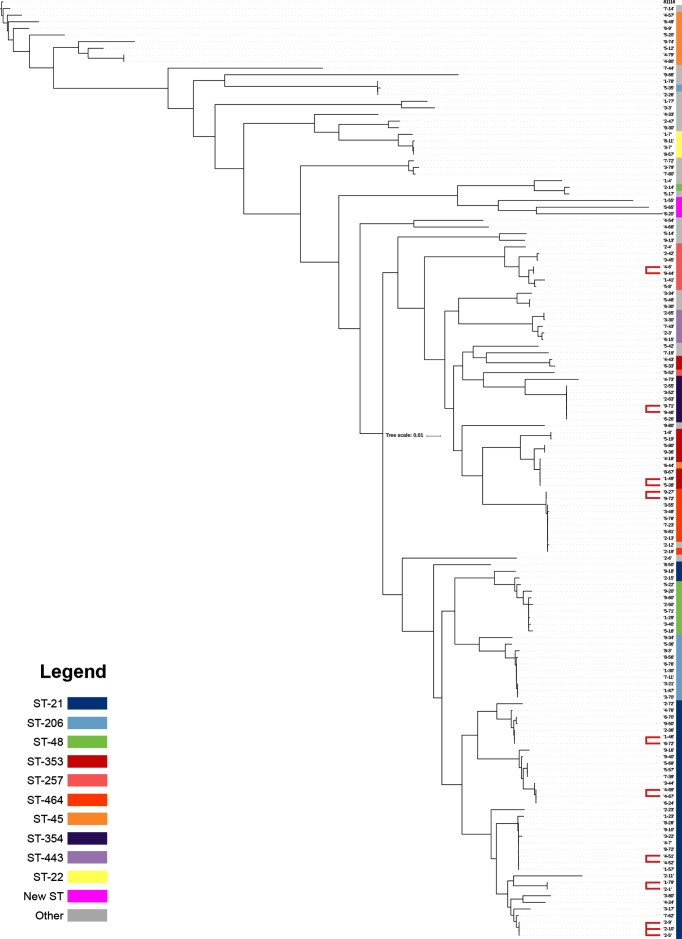
Core-genome phylogeny of 153 clinical *C. jejuni* isolates. The coloured bars represent the constituent CCs, and red lines indicate isolates that bear significant genetic homology (i.e. branch length=0) at the core-genome level. This tree is rooted to reference sequence 81116, resulting in a core-genome size of 115 596 bp generated using Parsnp [21] and iTOL [22]. Scale bar represents percentage of core genome size.

Three *C. jejuni* isolates (1-55, 5-65 and 6-20) were identified as novel sequence types. These isolates yielded full, unique allele profiles that did not belong to any previously assigned sequence type in the *Campylobacter* PubMLST database. These isolates form a sub-clade in the core-genome *C. jejuni* phylogeny (Fig. 3), branching from CC ST-573, which is associated with poultry [23]. These isolates were submitted to BigsDB under IDs 43837, 70894 and 70895, respectively, and are currently awaiting sequence-type assignment.

### Comparative genomics reveals epidemiological links between sporadic cases

**Fig. 4.**
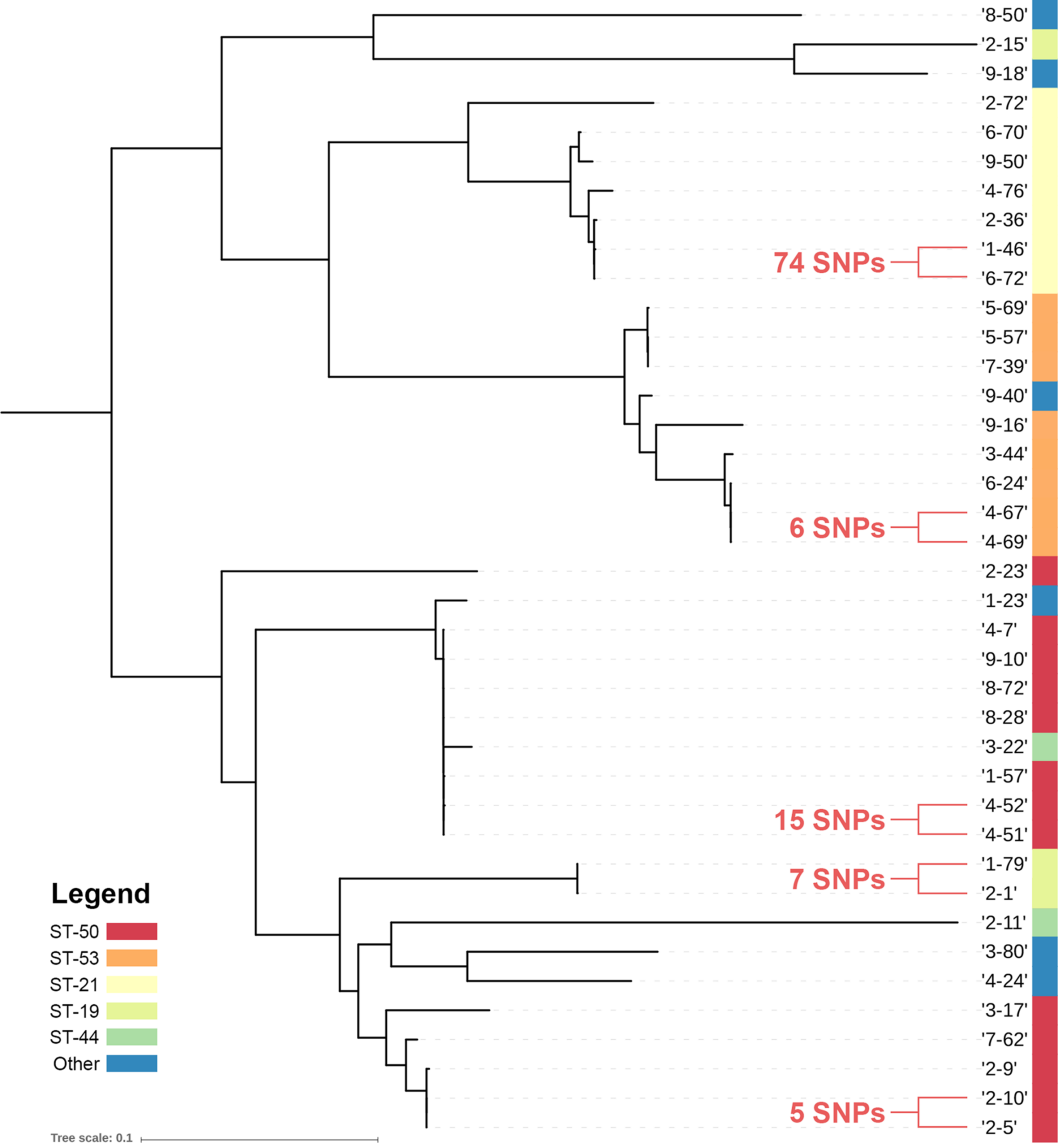
Core-genome phylogeny of isolates belonging to CC ST-21 with isolate pairs and respective number of SNPs highlighted. The tree is rooted to the mid-point with isolate 9-40 used as a reference and a core-genome size of 915 331 bp - scale bar represents percentage of core genome size. Sequence types with more than one constituent isolate are annotated with coloured bars. The figure was generated using Parsnp [21] and iTOL [22].

Separate core-genome phylogenies were reconstructed for all (*n*=153) *C. jejuni* isolates (Fig. 3) and *C. coli* isolates (data not shown). A separate core-genome phylogeny was also produced for CC ST-21 isolates (Fig. 4). Isolates that appeared to share a common source based on these phylogenies (i.e. branch length 0) were analysed using wgMLST and read-mapping approaches. Nine isolate pairs in total were considered to be potentially linked by these computational methods, which are listed in Table 1 and discussed below.

**Table 1.** Homology measured by wgMLST and mapping approaches of potentially linked *Campylobacter* spp. isolates

Isolates*	CC	Sequence type	Variable loci	Raw SNPs†	Filtered SNPs‡
2-5/2-10	ST-21	ST-50	1	5	1
4-69/4-67	ST-21	ST-53	35	9	6
2-1/1-79	ST-21	ST-19	20	20	7
9-71/9-46	ST-354	ST-354	16	11	11
4-52/4-51	ST-21	ST-50	19	34	15
1-49/5-38	ST-353	ST-5	44	40	36
1-46/6-72	ST-21	ST-21	49	63	74
2-9/2-10	ST-21	ST-50	35	152	110
4-6/9-44	ST-257	ST-257	42	370	50

*Reads from isolates on the right were mapped against *de novo* assemblies of isolates on the left, based on the isolate that contained the fewest identified loci.

†Number of SNPs with a mapping and base quality of ≥30, depth of ≥8 and present in more than 90 % of reads.

‡Number of SNP after removal of likely recombinant regions.

Upon further investigation, five genotypically clustered isolate pairs were found to contain fewer than 30 single nucleotide polymorphisms (SNPs), and shared a temporal association (isolated between 1 and 10 days apart, mean=3.6). Four of these genotypically similar isolate pairs were verified as belonging to separate patients and households. The closest relative isolates (2-5 and 2-10) yielded just one SNP. The position of SNPs identified within all isolate pairs and their effect on amino acid composition is displayed in Table S1 (available with the online version of this article).

We detected genotypically linked isolates in 2.5 %of the sampled population, whilst this supports previous observations that *Campylobacter* infection is typically sporadic [9], it demonstrates that genotypic linkage, and therefore potential same-source infections, can be observed at relatively minor levels. The frequency of genotypically linked cases may, however, be enhanced in the wider community. Only one in nine cases of campylobacteriosis will be reported at a national level as a result of a confirmed laboratory analysis [24]. The severity of campylobacteriosis symptoms is often highly variable, driven by factors such as host immunity and infectious dose [25], with milder manifestations of the disease contributing to the under-reported nature of the illness. The 2 .5% case linkage observed amongst this dataset, therefore, may be representative of a larger number of linked infections that do not generate a pathology sufficient for clinical consultation. Additionally, as the sequencing dataset only covers ~22 % of laboratory confirmed cases across Nottinghamshire, the detected linkage is approximately proportional to 1/45th of the potential total of *Campylobacter* infections, which is likely to have an impact on cross-comparability between other studies.

### Read mapping mitigates assembly errors for finer comparative resolution

Previous studies have relied on wgMLST as a comparative tool [9, 26], the strength of which lies in its originally described context (i.e. speed, simplicity, comparability). Whilst it is an excellent method for rapid analysis, our results show the importance of read mapping as a finer scale tool for genomic investigation. Using wgMLST alone, isolate pair 4-69/4-67 appears to be unrelated, with 35 variable loci between them (Table 1). Read mapping of the same isolate pair reveals just six SNPs. Further discordance was also observed between the wgMLST and read-mapping approaches. Amongst the 5 most homologous isolate pairs, 19 loci were found to contain variation by read mapping that did not contain any alternative alleles via wgMLST (Table S2). Furthermore, 85 loci yielded disparate alleles by wgMLST that were not detected via read mapping. Of these 85 loci, 9 were found to be variable amongst multiple isolate pairs (Table S2).

When considering the quality filtering imposed by the read-mapping approach, as well as the high mapping quality observed amongst called SNPs (mean=1466), it is likely that this disparity in identified variation is due to errors generated during the *de novo* assembly process. Loci that fail to correctly assemble will be unavailable to wgMLST comparison. This can be observed in the case of isolate pair 2-5 and 2-10, whereby locus CAMP1257 is not detected as variable via wgMLST due to truncation of the gene sequence in the assembly of isolate 2-10. Similarly, locus CAMP1064 in isolate pair 4-69 and 4-67 is located at the end of a contig in isolate 4-69, which also prevents comparison within this gene. Read mapping circumvents this issue, and instead is reliant on the accuracy and quality of the reference genome against which reads are mapped. Furthermore, wgMLST does not analyse intergenic regions – a total of six SNPs were detected at intergenic positions via mapping amongst the five most homologous isolate pairs (Table 1), providing an additional benefit to read-mapping approaches. To robustly investigate the observed differences in methodologies, future work may consider resequencing isolates with known homology.

### Repeat patient sampling reveals a SNP generated within 24 h

The most homologous isolate pair (2-5 and 2-10) was collected a day apart on the 24th and 25th of February, respectively (Table S2). These samples were obtained from the same patient, and revealed a SNP in the locus Cj1341c/CAMP1257, encoding the membrane protein Maf6 (Table 1). The *maf6* locus is one of several phase-variable genes within the *Campylobacter* genome and has been shown to come under variation within same-patient samples in other genomic studies [9, 20].

This polymorphism resulted in a non-synonymous alteration to the amino acid residue, which may modulate protein function and may reflect ongoing adaptation to the host. As the sample collection occurred on separate days, the variation observed may be a result of sampling an infectious population at different stages or progressions of the disease. Given the acute nature of campylobacteriosis and the level of variation observed amongst these isolates, it may suggest that *Campylobacter* is undergoing genetic variation within a single infection. Alternatively, the patient may have been infected with a mixed starting population. Additional study would be necessary to draw further conclusions.

### Conclusion

Using a read-mapping approach, case linkage is demonstrable amongst a dataset of presumably sporadic cases of campylobacteriosis. Whilst the relative rate of case linkage is low (2.5 %), in the wider community it may be enhanced due to the under-reported nature of campylobacteriosis and the 22 % representative sequenced sample. This study highlights the efficacy of read mapping as an objective measure of genetic homology, which offers a robust resolution of individual isolate pairs and avoids errors introduced via errors in the *de novo* assembly.

## Data bibliography

1. Dunn S, Pascoe B, Turton J, Fleming V, Diggle M, McNally A, Manning G. Sequencing Read Archive, BioProject Accession PRJNA420922 (2017).

## Supplementary Data

Supplementary File 1
